# Molecular Stability as a Translational Gate: A Structured Framework for Target Validation in Genetic Cardiomyopathy

**DOI:** 10.7759/cureus.108722

**Published:** 2026-05-12

**Authors:** Sanghati Basu, Mahesh Narayan, Prakash Narayan

**Affiliations:** 1 Healthcare Informatics, University of Illinois Springfield, Springfield, USA; 2 Chemistry and Biochemistry, The University of Texas at El Paso, El Paso, USA; 3 Bioinformatics, Nodes and Edges LLC, Raleigh, USA

**Keywords:** biomarkers, concordance scoring, cross-cohort reproducibility, dilated cardiomyopathy, hypertrophic cardiomyopathy, molecular stability, multi-omics, sarcomere, target validation, translational framework

## Abstract

The dominant translational error in genetic cardiomyopathy is the treatment of pathogenicity annotation and mechanistic plausibility as jointly sufficient for clinical advancement, absent evidence that a target’s molecular consequences are both stable and reproducible across independent patient cohorts. This perspective argues that molecular stability is a function of mechanism and not of genetic evidence and that cross-cohort concordance must serve as an explicit development gate alongside mechanistic plausibility. We synthesize published evidence across sarcomeric biology, calcium signaling, fibrosis, metabolic remodeling, and immune crosstalk in hypertrophic cardiomyopathy and dilated cardiomyopathy and critically evaluate how biological heterogeneity, incomplete penetrance, and model limitations introduce translational risk that currently goes unquantified. Evidence is organized by mechanistic domain and evaluated for reproducibility strength using a structured synthesis approach. A seven-step translational framework is proposed, operationalized through a five-domain Molecular Concordance Scoring Matrix. The matrix is presented as one implementation of the broader principle that stability must be demonstrated, not assumed, and is the binding contribution. An illustrative evidence map for representative cardiomyopathy targets is provided, with expected concordance tiers grounded in published mechanistic evidence. This framework identifies cardiomyopathy as an exemplar of a broader problem in genetically anchored but molecularly heterogeneous disease and specifies the empirical agenda required to validate and generalize it.

## Introduction and background

Hypertrophic cardiomyopathy (HCM) and dilated cardiomyopathy (DCM) represent two of the most common inherited cardiac disorders and are major contributors to heart failure, arrhythmia, and sudden cardiac death worldwide. HCM affects approximately one in 500 individuals globally, though contemporary genetic screening studies suggest prevalence may be closer to one in 200 when genotype-positive individuals are included. DCM has an estimated prevalence of approximately 1 in 250-500 individuals and accounts for a substantial proportion of nonischemic heart failure and heart transplantation cases. In the United States, cardiomyopathies collectively contribute to hundreds of thousands of heart failure hospitalizations annually, with heart failure-related healthcare expenditures exceeding $30-40 billion per year, a portion of which is attributable to inherited and nonischemic cardiomyopathies. Globally, heart failure and cardiomyopathy-related conditions represent a major and growing healthcare burden, with worldwide heart failure costs estimated to exceed $100 billion annually [1]. Despite advances in genetic diagnosis and mechanistic understanding, cardiomyopathies remain a major cause of morbidity, mortality, and healthcare expenditure, highlighting the need for improved translational frameworks to move mechanistic discoveries into durable therapeutic strategies. Importantly, the chasm between genetic clarity and translational success in these diseases represents both a scientific and a socioeconomic problem.

HCM and DCM are frequently described as monogenic disorders, but penetrance and expressivity vary substantially even among carriers of the same primary mutation [2]. The dominant translational error in genetic cardiomyopathy is the treatment of pathogenicity annotation and mechanistic plausibility as jointly sufficient for clinical advancement, absent evidence that a target’s molecular consequences are stable and reproducible across independent patient cohorts. Pathogenic variants in sarcomeric genes such as ß-myosin heavy chain (MYH7) and myosin-binding protein C (MYBPC3) account for the majority of HCM cases [3], whereas titin (TTN) truncating variants are amongst the most common contributors to DCM [4]. While sarcomere-modulating agents have demonstrated clinical efficacy in obstructive HCM [5], many targets grounded in plausible signaling biology have stalled in development or yielded inconsistent clinical signals. The persistent gap between mechanistic discovery and therapeutic durability is not adequately explained by insufficient preclinical work; it reflects a structural assumption that has gone unexamined. That assumption is that a variant classified as pathogenic in ClinVar [6] will produce consistent downstream molecular perturbations across independent patient cohorts, disease stages, and biological contexts. Pathway activation states, fibrotic burden, metabolic remodeling, and inflammatory signatures differ across cohorts stratified by age, hemodynamic load, comorbidities, or therapeutic exposure [7]. The same is true of high-dimensional multi-omics signals: transcriptomic and exosomal biomarker profiles identified in a single cohort are highly sensitive to pre-analytic variation and pipeline differences [8], and absent explicit cross-cohort validation, they remain cohort-specific rather than disease-defining.

Herein, we posit that target validation in genetic cardiomyopathy must extend beyond variant association and mechanistic plausibility to include systematic evaluation of cross-cohort reproducibility, biomarker stability, model fidelity, and modality alignment. We conducted a structured translational evidence synthesis with systematic search elements. The objective was not to generate pooled effect estimates but to critically evaluate translational evidence across mechanistic, biomarker, and therapeutic domains in genetic cardiomyopathies, with emphasis on studies incorporating mechanistic mapping, reproducibility considerations, and clinical or development relevance. Evidence was organized by mechanistic domain and evaluated using graded language, supported, suggestive, limited, or not yet established, to reflect the strength of available cross-cohort reproducibility data rather than the simple presence or absence of mechanistic evidence.

## Review

Methods

A structured query was performed across PubMed/MEDLINE, Web of Science, Scopus, Embase, and ClinicalTrials.gov. Search terms included combinations of “hypertrophic cardiomyopathy”, “dilated cardiomyopathy”, “sarcomere mutation” specific gene identifiers (MYH7, MYBPC3, and TTN), key signaling pathway terms such as calcineurin-nuclear factor of activated T-cells (NFAT), cardiac remodeling, and fibrosis; multi-omics platforms such as proteomics, metabolomics, and exosomes and translational concepts such as reproducibility, molecular stability, biomarker qualification, and genome editing. The initial search identified approximately 1,840 potentially relevant records. Following title and abstract screening for mechanistic or translational relevance, 312 full-text articles were assessed for eligibility. Of these, 18 were selected as primary citations based on mechanistic specificity, human validation, cross-cohort relevance, or direct therapeutic significance. Earlier seminal studies were included, as they were foundational to established mechanistic understanding.

Studies were categorized into six thematic domains: genetic architecture and variant interpretation; mechanistic signaling pathways; fibrotic and remodeling biology; biomarker and multi-omics integration; therapeutic modality development; and reproducibility and translational risk. Within each domain, evidence was evaluated for mechanistic support strength, human validation, cross-cohort reproducibility, model fidelity, and therapeutic alignment. Emphasis was placed on mechanistic completeness and translational clarity rather than citation frequency. Publication bias was acknowledged as a limitation, given the likelihood that positive mechanistic findings are overrepresented in the published literature. The concordance tier assignments presented represent expected tiers grounded in published mechanistic evidence, not original computed scores. This review follows a narrative synthesis approach with systematic search elements and does not adhere to the PRISMA guidelines, as no pooled quantitative analysis was performed.

Mechanistic architecture of genetic cardiomyopathies

HCM arises predominantly from gain-of-function sarcomeric mutations, with variants in MYH7 altering ATPase cycling and force generation efficiency, while MYBPC3 mutations cause haploinsufficiency or thick filament destabilization [9,10]. TTN truncations in DCM compromise sarcomeric elasticity and mechanosensing, impairing mechanotransduction pathways that regulate remodeling programs [4]. Both diseases converge on altered biomechanical signaling despite divergent phenotypic expression, establishing distinct but partially overlapping translational profiles.

Sarcomeric dysfunction propagates into dysregulated calcium homeostasis, activating calcineurin-NFAT signaling and intersecting with mitogen-activated protein kinase (MAPK) cascades, phosphoinositide 3-kinase-protein kinase B pathways, and mitochondrial stress responses [7,11]. These signals form a network rather than a linear chain. Targeting any single node may yield variable results depending on disease stage, genetic background, and comorbidities, precisely the instability the framework is designed to detect before development commitments are made.

Myocardial fibrosis emerges early in HCM, contributing to ventricular stiffness and arrhythmogenic substrate formation [12]. Circulating markers such as galectin-3 and ST2 reflect global remodeling rather than mutation-specific mechanisms [13,14], making fibrosis more suitable as a progression marker than a primary therapeutic target. Across calcium signaling, metabolic remodeling, and inflammatory pathways, activation states vary substantially across patient cohorts, introducing the translational instability that motivates the concordance framework (Table 1).

**Table 1:** Comparative translational profiles of HCM and DCM EXPLORER-HCM [5] Stability classifications reflect the synthesis of evidence described in the “Mechanistic architecture of genetic cardiomyopathies,” “Translational challenges in genetic cardiomyopathy,” and “Reproducibility, molecular stability, and development risk” sections. Ca2+, calcium; DCM, dilated cardiomyopathy; HCM, hypertrophic cardiomyopathy; LMNA, lamin A/C; RBM20, RNA-binding motif protein 20; SCN5A, sodium voltage-gated channel alpha subunit 5.

Domain	HCM	DCM
Primary genetic drivers	MYH7, MYBPC3 (sarcomeric; gain-of-function/haploinsufficiency)	TTN truncations; LMNA, SCN5A, RBM20
Proximal functional defect	Hypercontractility; increased cross-bridge duty ratio; enhanced Ca2+ sensitivity	Impaired force generation; cytoskeletal instability; mechanosensing failure
Mechanistic chain clarity	High - mutation to hypercontractility to obstruction relatively direct	Moderate-low - broader multi-pathway remodeling cascade
Penetrance	Variable; strongly modulated by common genetic variants and lifestyle factors [2]	Incomplete; environmental triggers (myocarditis or pregnancy) play a major role [15]
Fibrosis profile	Interstitial and replacement; can appear early; regionally variable [12]	Progressive dilation-associated; late-stage dominant
Molecular stability (pathway level)	Higher for proximal sarcomeric targets; lower for downstream remodeling	Generally lower; context-dependent pathway activation across cohorts
Therapeutic success example	Mavacamten (EXPLORER-HCM, [5]); targets hypercontractility directly	No equivalent proximal target; neurohormonal agents standard; gene therapy investigational
Preferred translational strategy	Upstream sarcomere modulation; phenotype-proximal targeting	Systems-level stabilization; remodeling control; multi-target approaches

Translational challenges in genetic cardiomyopathy

The mechanistic heterogeneity of HCM and DCM confers translational risk. Even within families carrying identical sarcomeric mutations, left ventricular wall thickness, fibrosis burden, and arrhythmic risk vary substantially because common modifier variants and environmental exposures shape expressivity [2]. Molecular signatures derived from one cohort may therefore reflect context-specific biology rather than universal disease drivers. Standard biomarkers such as BNP and NT-proBNP reflect hemodynamic stress but lack mechanistic specificity [16]; exosomal biomarkers offer resolution but are highly sensitive to isolation protocols and batch effects [8]. Model systems compound the problem: rodent hearts express predominantly alpha-myosin heavy chain rather than MYH7 [10], and induced pluripotent stem cell-derived cardiomyocytes (iPSC-CMs) remain immature without rigorous maturation protocols [17] and lack the multicellular fibroblast-endothelial-immune architecture that drives remodeling dynamics [18]. Each of these instabilities, biological, biomarker-level, and model-level, is a domain within the concordance scoring framework, making their explicit evaluation a mandatory rather than optional component of target validation.

Reproducibility, molecular stability, and development risk

Clinical Annotation Is Not Molecular Validation

A pathogenic classification in ClinVar indicates accumulated clinical and genetic evidence supporting disease association [6]. It does not guarantee that the gene’s downstream molecular phenotype will manifest consistently across independent cohorts, tissue samples, disease stages, or environmental contexts. The assumption that clinical pathogenicity implies molecular stability has been implicitly embedded in many translational programs. When this assumption is not examined directly, it introduces development risk that is rarely quantified but frequently realized.

Sarcomeric genes such as MYH7 and MYBPC3 have strong genetic credibility in HCM [3,9]. TTN truncations are well established in DCM [4]. These associations are robust at the genetic level. However, the transcriptomic, proteomic, and signaling consequences of these variants are not always consistent across datasets. Cohort composition, disease stage, ventricular loading conditions, medication exposure, and comorbidities all shape molecular readouts. Even fibrosis shows temporal variability, appearing early in some individuals but varying in magnitude and spatial distribution across patients and disease duration [12]. This variability is not statistical noise but reflects biological heterogeneity that must be explicitly characterized rather than explained away.

Harper et al. [2] demonstrated that common genetic variants and modifiable risk factors influence HCM expressivity, meaning that even within a shared primary sarcomeric mutation, the broader genetic and environmental background modulates phenotype. When downstream molecular signatures are compared across cohorts without accounting for this variability, instability is often misinterpreted as technical inconsistency rather than recognized as biological context dependence, a misclassification with significant implications for development strategy.

Molecular Concordance as a Translational Gate

Reproducibility across independent human datasets should function as an explicit translational gate, applied before advancement into Investigational New Drug (IND)-enabling programs. Three measurable components define concordance: directional agreement across cohorts, effect size similarity, and statistical reproducibility after multiple testing correction. A fourth component, baseline interindividual variability, must be benchmarked against healthy tissue. The GTEx Consortium [19] demonstrated that cardiac gene expression variability differs substantially across genes, with some exhibiting tight normal distributions and others varying widely across individuals. A pathway signal exceeding expected baseline variability carries greater translational weight than one falling within physiological dispersion.

Concordance is graded rather than binary and produces three actionable decision categories. A target demonstrating strong directionality, consistent effect size, and statistical reproducibility across multiple independent cohorts supports an advanced decision: progression to IND-enabling studies is warranted. A target with directional agreement but inconsistent effect size or significance supports a Conditional Advance: progression requires a prospective validation plan specifying additional cohorts, standardized biomarker assays, or genotype-stratified analyses before phase II initiation. A target exhibiting inconsistent direction across cohorts warrants a Do Not Advance decision: orthogonal validation from independent human datasets is required before re-entry into development consideration. These three categories, such as Advance, Conditional Advance, and Do Not Advance, are the operational outputs of the scoring matrix described in the “A structured translational framework for genetic cardiomyopathy development” section. Their explicit pre-specification before data are examined prevents post hoc rationalization of advancement decisions.

Development Risk Categories

Drug development programs implicitly assume stability at several levels: that the target is causally upstream of disease phenotype; that modulating the target will shift downstream biology; that downstream biology can be measured reliably; and that measurable signals correlate with clinical improvement. Instability at any of these levels increases risk, yet instability is rarely quantified early in development. Sarcomere modulation provides an instructive contrast. Small-molecule myosin inhibitors target contractile mechanics directly, bypassing some upstream transcriptional variability. The clinical efficacy of mavacamten in obstructive HCM [5] illustrates how a phenotype-proximal target can succeed even when upstream signaling cascades vary. The mechanism is tightly linked to hypercontractility, a relatively stable functional phenotype. Targets positioned further upstream in signaling cascades, such as calcineurin-NFAT or MAPK nodes, may exhibit pronounced context dependence [7,11]. Metabolic remodeling represents a parallel example: altered fatty acid oxidation and mitochondrial energetic stress are well documented in HCM myocardium [20], but their cross-cohort reproducibility is limited by dependence on disease stage, comorbid metabolic syndrome, and pharmacotherapy exposure. Without cross-cohort validation, distinguishing causal from reactive signaling becomes difficult. Concordance assessment becomes in this context a mechanism of risk control, forcing early confrontation with heterogeneity rather than deferring instability to late-phase trials where the cost of failure is highest.

Current Evidence Map for Concordance by Mechanism Class

A structured review of published mechanistic evidence reveals a pattern consistent with the framework’s central prediction: transcript-level reproducibility is not uniformly distributed across ClinVar-annotated cardiomyopathy genes but tracks with pathogenic mechanism class. The following synthesis organizes available published evidence using graded language to reflect actual evidentiary strength rather than assumed concordance. Sarcomeric HCM genes (MYH7, MYBPC3, and TNNT2) exhibit relatively robust genetic anchoring, supported by decades of segregation analysis, functional studies, and population frequency data [3,9,10]. Their pathogenic mechanism, however, operates predominantly at the protein level, viz., altered ATPase kinetics, cross-bridge cycling efficiency, and thick filament regulation [10,21]. Published transcript-level evidence across independent cohorts is limited and context-dependent: studies report variable and often opposing directions of differential expression depending on disease stage, hypertrophic load, and cohort composition [2,19]. Secondary upregulation of sarcomeric transcripts as a structural compensation for hypertrophic stress is well described [21], making it difficult to separate variant-driven perturbation from remodeling response in RNA-level data. Cross-cohort transcript reproducibility for this gene class should therefore be considered not yet established.

TTN truncation in DCM occupies a distinct position: genetic credibility is robust [4], and mechanosensing failure is mechanistically grounded, but the downstream transcriptomic consequences are suggestive rather than supported across multiple independent cohorts. Incomplete penetrance [15] and dependence on environmental triggers mean that cohorts enriched for recently symptomatic patients show different pathway activation profiles than those including subclinical mutation carriers. Published transcript-level evidence is largely pathway-level and remodeling-associated, not titin-specific, and cross-cohort reproducibility of any specific molecular signature in this context should be considered limited pending formal multi-cohort analysis.

RNA-processing DCM genes (RNA-binding motif protein 20 (RBM20) and lamin A/C (LMNA) represent the strongest conceptual candidates for transcript-level concordance. RBM20 variants directly impair pre-mRNA splicing of titin and other cardiac transcripts, producing aberrant isoforms detectable at the RNA level [22]. LMNA variants disrupt nuclear structural integrity with documented downstream effects on cardiac gene expression programs [15]. Both genes operate at or close to the transcript level, making mechanism-proximal RNA signatures more plausible than for contractile sarcomeric genes. This principle extends to non-coding RNA regulatory networks: microRNA-208a, encoded within MYH6 and regulating cardiac hypertrophy and conduction, exemplifies how a transcript-level regulatory mechanism produces a reproducible molecular perturbation directly measurable at the RNA level [23]. Published evidence is suggestive of higher transcript-level reproducibility for this mechanism class broadly, though formal cross-cohort concordance quantification for individual genes remains to be established. Their expected concordance tier is higher than for sarcomeric or TTN genes, making them appropriate positive-control candidates for any empirical concordance evaluation. This evidence map is summarized in Table 2, which presents an illustrative application of the framework to representative targets. Tier assignments are graded expectations based on published mechanistic evidence, not computed scores.

**Table 2:** Illustrative application of the framework to representative cardiomyopathy targets Concordance tier assignments represent expected tiers grounded in published mechanistic evidence, not original computed scores. Language reflects evidence strength: supported, suggestive, limited, or not yet established. Expected concordance tier is grounded in published mechanistic evidence as synthesized in the “Current evidence map for concordance by mechanism class” section; it is not a computed score. Graded language throughout: supported = strong published evidence across multiple independent sources; suggestive = mechanistically plausible with partial evidence; limited = heterogeneous or insufficient evidence; not yet established = evidence gap identified.

Gene/disease	Genetic credibility	Mechanism level	Expected molecular readout	Published cross-cohort evidence
MYH7/HCM	Supported [3,9]	Protein (ATPase kinetics)	Transcript	Limited, context-dependent; secondary upregulation documented [10,21]
MYBPC3/HCM	Supported [3,9]	Protein (haploinsufficiency)	Transcript	Limited, variable direction across cohorts; remodeling contribution confounds [10,21]
TTN trunc/DCM	Supported [4]	Protein/cytoskeletal (mechanosensing)	Transcript (indirect)	Suggestive, pathway-level remodeling evidence; titin-specific transcript signal limited [4,15]
RBM20/DCM	Supported [15,22]	RNA (splicing regulation)	Transcript (direct)	Suggestive of higher reproducibility; aberrant isoforms measurable by RNA-seq [22]
LMNA/DCM	Supported [15]	Regulatory/nuclear (gene expression programs)	Transcript (mechanism-proximal)	Suggestive, downstream expression effects documented; cross-cohort quantification not yet established [15]
TNNT2/HCM-DCM	Supported [3]	Protein (sarcomeric)	Transcript	Limited, phenotype-dependent; bi-directional expression changes reported [10]

Multi-omics integration and AI: opportunity and methodological fragility

Multi-omics and AI-driven discovery expand the candidate target landscape but introduce fragility that the concordance framework is specifically designed to detect. High dimensionality increases the risk of overfitting and dataset-specific artifacts [24]. When a candidate compound or target demonstrates mechanistic activity across multiple pathways simultaneously, as in the case of epigallocatechin gallate, active across transforming growth factor-beta fibrosis, sarcomeric calcium sensitivity, and inflammatory signaling [25], mechanistic specificity collapses. It becomes impossible to identify which mechanism drives any clinical effect, design a reproducible pharmacodynamic biomarker, or predict responder subgroups. Multi-target plausibility without cross-cohort concordance remains insufficient grounds for development advancement. Pre-analytic variability represents an underappreciated vulnerability. Proteomic and exosomal analyses are sensitive to sample handling, isolation protocols, batch effects, and normalization [8]. GTEx data demonstrate that disease-associated perturbations must be interpreted relative to normal interindividual cardiac gene expression variance [19] viz. a gene with moderate fold-change in a single cohort may fall within physiological dispersion population-wide. Metabolomic profiling of HCM and DCM myocardium has revealed altered energetic remodeling signatures [20], but cross-cohort reproducibility is limited by metabolic comorbidity burden and pharmacotherapy exposure. Algorithmic sophistication must not mask biological fragility.

Comparative translational profiles: HCM vs. DCM

HCM and DCM exhibit distinct translational profiles with direct implications for concordance scoring and target selection (Table 1). HCM’s mechanistic chain, from sarcomeric mutation to hypercontractility to outflow tract obstruction, is relatively direct and phenotype-proximal, a property that likely contributed to the therapeutic success of mavacamten [5]. DCM reflects broader structural instability driven by TTN truncations impairing mechanosensing and cytoskeletal integrity [4,15], with therapeutic targets distributed across a less stable signaling landscape. These differences argue for disease-specific concordance evaluation rather than shared translational logic across the two disease programs.

Worked Example: Applying the Framework to TTN-Truncation in DCM

TTN truncating variants are among the most robustly established genetic contributors to DCM, identified in approximately 25% of familial and 18% of sporadic cases [4]. Large-scale sequencing programs have effectively separated disease-associated truncations from benign population-frequency variants. Under the framework, genetic credibility (C4) would meet the highest category: direct ClinVar P/LP linkage with functional evidence.

The causal sequence from TTN truncation through sarcomeric elasticity compromise to mechanosensing failure is mechanistically grounded. TTN’s role in Z-disc organization, passive tension generation, and mechanotransduction signaling is well characterized. However, the chain becomes inferential at the transition from cytoskeletal instability to downstream inflammatory activation, neurohormonal engagement, and progressive chamber dilation. These secondary remodeling steps are not titin-specific; they represent a shared final common pathway activated by diverse upstream insults. A therapeutic targeting TTN-specific mechanosensing must therefore demonstrate that its mechanism of action operates upstream of this convergence, not within the shared remodeling cascade.

This is where TTN-related DCM earns a moderate rather than high concordance profile. While the genetic signal is stable, the transcriptomic and proteomic consequences of TTN truncation vary substantially across cohorts. Incomplete penetrance means that mutation carriers may remain clinically silent for decades. Environmental triggers, such as viral myocarditis, sustained hemodynamic overload, and pregnancy, interact with the genetic predisposition to determine disease expression [15]. Cohorts enriched for recently symptomatic patients will show different pathway activation profiles than those including preclinical carriers. Directional agreement across cohorts (C1) is moderate; effect size consistency (C2) is limited by this contextual variability; baseline variability ratio (C3) depends critically on disease stage and comorbidity burden. Under the proposed framework, TTN-related DCM would be expected to fall within a moderate concordance range, supporting a conditional-advance interpretation pending formal multi-cohort validation.

Circulating TTN fragments have been investigated as damage markers in DCM, but their pharmacodynamic utility as indicators of target engagement for a TTN-directed therapeutic remains incompletely characterized. Mechanical stretch-responsive biomarkers and Z-disc integrity markers represent candidate pharmacodynamic tools but require standardized assay development and cross-site reproducibility validation before serving as trial enrichment or response endpoints.

iPSC-derived cardiomyocytes carrying TTN truncations can replicate mechanosensing failure and reduced passive force generation [17]. However, the progressive dilation and fibrotic remodeling that characterize human TTN-related DCM require multicellular architecture, such as fibroblasts, endothelial cells, and macrophages, that isolated cardiomyocyte systems cannot reproduce. Model recapitulation (C5) would meet only the moderate category under the framework: single-system validation with partial phenotypic alignment. Confirmation in engineered cardiac tissue or organoid systems with multicellular composition is required before the highest model-fidelity category can be satisfied.

Strategic conclusion - conditional advance: The TTN-DCM concordance profile argues for a conditional advance strategy: progression is warranted given strong genetic credibility but must be contingent on a prospective validation plan that includes real-world evidence (RWE)-based phenotypic stratification to identify the subpopulation with active pathway engagement, stage-specific enrollment criteria that control for the environmental trigger variability identified in Step 3, and biomarker qualification for at least one mechanistically anchored pharmacodynamic readout before phase II initiation. This approach applies the framework’s core principle: genetic credibility establishes the starting point, but molecular concordance determines the conditions under which advancement is responsible.

Worked Example: MYH7 and MYBPC3 as Genetically Anchored but Transcript-Unstable Targets

Genetic credibility (C4): MYH7 and MYBPC3 carry the strongest genetic credibility of any HCM loci, accounting for approximately 40% of genotype-positive HCM cases [3,9]. Segregation data, population frequency analyses, and functional studies in model systems have established these genes as unambiguous causal contributors. Under the framework, genetic credibility (C4) would meet the highest category for both genes.

Mechanistic chain (C2 consideration): Pathogenic variants in these genes predominantly alter protein structure and contractile function, such as ATPase cycling rate, cross-bridge duty ratio, and thick filament regulatory dynamics, rather than transcript abundance. The downstream molecular phenotype in myocardium reflects hypercontractility and energetic stress, not consistently disrupted RNA-level expression of the mutant gene itself [10,21]. Sarcomeric gene expression in hypertrophied myocardium may, in fact, increase as a structural compensation, independent of variant-driven disruption [21].

Cross-cohort concordance (C1, C2, and C3): This mechanistic architecture has direct concordance implications. Transcript-level signatures of MYH7 and MYBPC3 across independent HCM cohorts are expected to show variable direction and magnitude of differential expression, depending on the relative contribution of hypertrophic upregulation versus variant-specific downregulation in each cohort’s composition. Direction agreement (C1) is therefore limited; effect size consistency (C2) is further constrained by stage-dependent secondary upregulation; and the baseline variability ratio (C3) may not clearly separate disease signal from normal interindividual variation as benchmarked against GTEx left ventricular profiles [19].

Translational implication: The strong genetic credibility of MYH7 and MYBPC3 makes them credible development anchors at Steps 1 and 2. However, their transcript-level concordance profile is expected to be moderate to low, consistent with their predominantly protein-level mechanism. This does not diminish their therapeutic relevance: mavacamten’s success at directly targeting hypercontractility confirms their importance [5]. Rather, it underscores the principle that genetic anchoring does not imply transcript-level biomarker stability. Development programs built on RNA-level signatures of these genes should treat transcript concordance as an unverified assumption requiring explicit multi-cohort validation before trial enrichment strategies are built around it.

Worked Example: RBM20 and LMNA as Transcript-Proximal Mechanism Candidates

Genetic credibility (C4): RBM20 encodes an RNA-binding protein that regulates the alternative splicing of titin and several other cardiac transcripts. Pathogenic variants, predominantly in the RNA recognition motif domain, cause DCM with high penetrance and an often severe arrhythmic phenotype [15]. LMNA encodes Lamin A/C, a nuclear envelope structural protein; pathogenic variants cause laminopathic DCM, characterized by early atrioventricular conduction disease and a high sudden death risk. Both genes have well-established ClinVar P/LP classifications with multi-submitter review evidence, and both would meet the framework’s highest genetic-credibility category.

Mechanistic chain and transcript-level mechanism: The mechanistic distinction from sarcomeric genes is important here. RBM20 variants directly impair pre-mRNA splicing of titin and other regulatory transcripts, producing aberrant isoforms that are measurable at the RNA level [22]. LMNA variants disrupt nuclear structural integrity and mechanotransduction signaling, with documented downstream effects on gene expression programs governing cardiomyocyte stress responses [15]. In both cases, the primary pathogenic mechanism is plausibly transcript-proximal: the molecular consequences are expected to manifest in RNA-level signatures in a more mechanism-direct manner than is the case for sarcomeric contractile genes.

Concordance expectation (C1, C2, and C3): For RBM20 and LMNA, directional agreement in differential expression across independent DCM cohorts is expected to be higher than for TTN truncation or sarcomeric HCM genes, because the primary mechanism of pathogenicity operates at or close to the transcript level. The baseline variability ratio (C3) is expected to be more favorable as well, since the disease signal reflects a mechanism-proximal perturbation rather than a secondary compensatory response. This does not mean concordance will be high by default: cohort composition, disease stage, and medication exposure still introduce variability. However, the mechanistic architecture of these genes makes them better candidates for transcript-based biomarker strategies than sarcomeric contractile genes.

Translational implication: RBM20 and LMNA represent a positive control stratum for concordance assessment: if a cross-cohort concordance framework cannot identify higher stability for mechanism-proximal RNA-level targets than for protein-mechanism sarcomeric genes, the framework lacks discriminative power. Their inclusion as a reference stratum in any empirical concordance evaluation would allow the scoring instrument to be calibrated against biologically motivated expectations. The concordance profiles described across the three worked examples are summarized in Table 3, illustrating how mechanism class determines expected transcript stability and matrix recommendation for representative cardiomyopathy targets.

**Table 3:** Summary of concordance matrix recommendations for representative cardiomyopathy targets Transcript stability and matrix recommendations are grounded in published mechanistic evidence as synthesized in the “Worked example: applying the framework to TTN-truncation in DCM,” Worked example: MYH7 and MYBPC3 as genetically anchored but transcript-unstable targets,” and “Worked example: RBM20 and LMNA as transcript-proximal mechanism candidates” sections; they are not original computed scores. Translational status reflects expected cross-cohort concordance based on mechanism class. Matrix Recommendation corresponds to the three decision categories defined in the “Molecular concordance as a translational gate”: Advance (score 8-10), Conditional Advance (score 5-7), and Do Not Advance (score below 5). DCM, dilated cardiomyopathy; HCM, hypertrophic cardiomyopathy; P/LP, pathogenic or likely pathogenic

Target	Genetic profile	Transcript stability	Translational status	Matrix recommendation	Section reference
TTN-truncation (DCM)	High genetic credibility; ~25% familial, ~18% sporadic DCM	Low/variable - protein-level mechanism; transcript signal indirect	Moderate concordance; context-dependent across cohorts	Conditional Advance	Worked example: applying the framework to TTN-truncation in DCM
MYH7/MYBPC3 (HCM)	Highest genetic credibility; ~40% genotype-positive HCM cases	Unstable - protein-level ATPase/contractile mechanism; transcript direction variable	Limited cross-cohort concordance; secondary upregulation confounds signal	Conditional Advance (transcript biomarker strategy requires multi-cohort validation)	Worked example: MYH7 and MYBPC3 as genetically anchored but transcript-unstable targets
RBM20/DCM	High penetrance; ClinVar P/LP; RNA recognition motif variants	High - RNA splicing mechanism; aberrant isoforms directly measurable	Higher concordance expected; mechanism-proximal transcript signal	Advance (positive control stratum)	Worked example: RBM20 and LMNA as transcript-proximal mechanism candidates

A structured translational framework for genetic cardiomyopathy development

Target validation in cardiomyopathy often follows a familiar trajectory: variant identification, pathway implication, model-based confirmation, and therapeutic hypothesis generation. What is frequently absent is a structured evaluation of reproducibility, mechanistic completeness, and development risk before advancement into costly translational programs. The following seven-step framework addresses these gaps.

**Figure 1:**
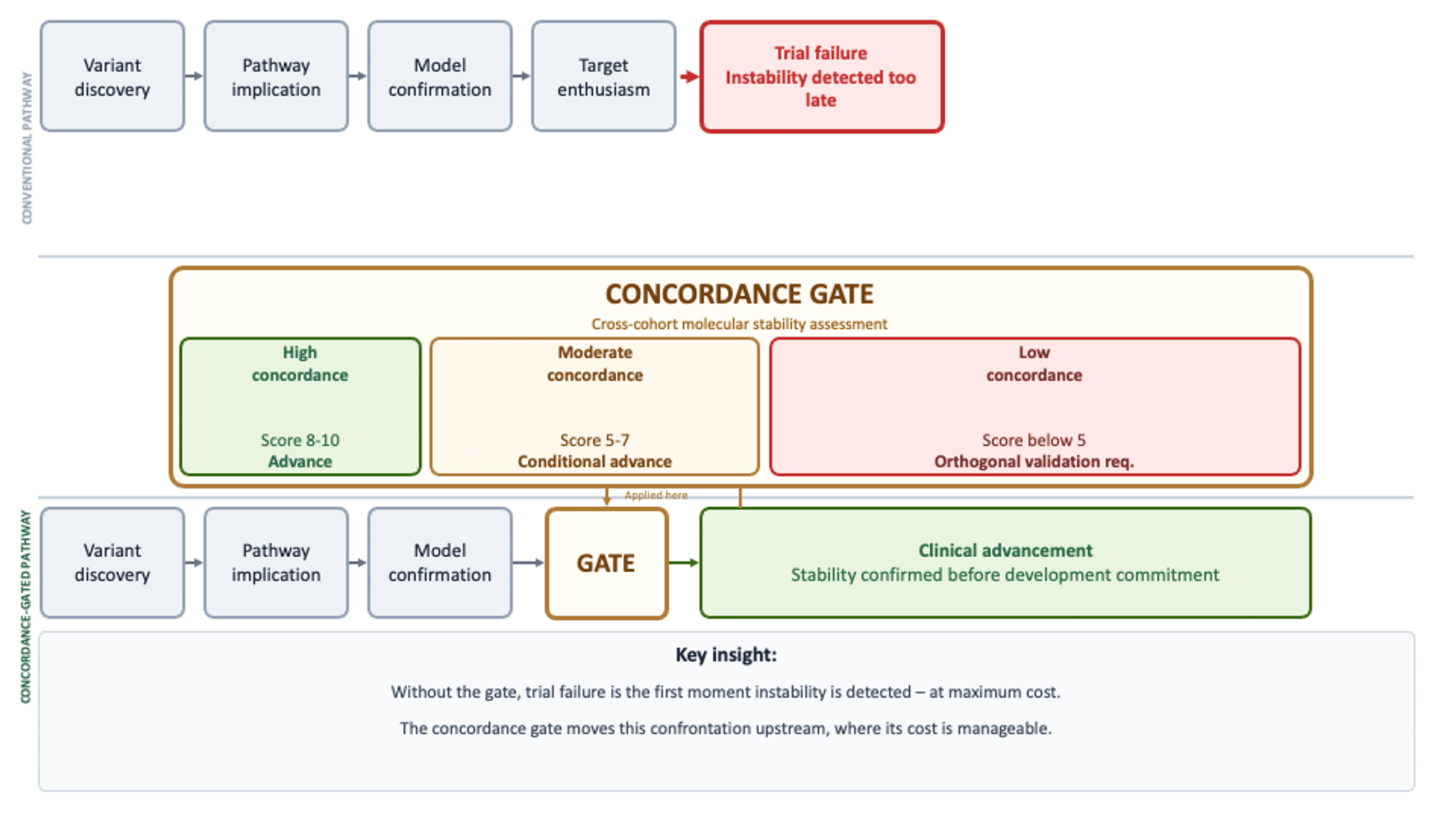
Why promising cardiomyopathy targets fail in translation The upper panel shows the translational failure pathway without the concordance gate: variant discovery, pathway implication, model confirmation, and target enthusiasm all proceed to trial failure, the first moment at which molecular instability is detected. The lower panel shows the pathway with the gate inserted after model confirmation. Targets scoring 8-10 advance unconditionally; scores of 5-7 trigger conditional advance with a prospective validation plan; scores below 5 require orthogonal validation before reentry. The gate moves confrontation with instability upstream, where the cost of confrontation is manageable. This figure was created by the authors using Microsoft PowerPoint (Microsoft Corporation, Redmond, WA, USA).

The framework is offered as one implementation of a broader translational principle: stability should be treated as a mechanistic property and a development gate, not as a statistical afterthought. The matrix operationalizes that principle in a scored, documentable form. Other implementations are possible: the conceptual contribution is the principle itself, not the specific scoring instrument. The position of the concordance gate within the translational failure pathway is illustrated in Figure 1.

Confirm variant-disease association through robust genetic evidence, segregation analysis, population frequency assessment, and functional validation [3,6]. For DCM, TTN truncations provide a model of strong genetic linkage that separates benign from disease-associated variants [4]. Genetic credibility establishes the starting point but does not specify the therapeutic target or confirm downstream molecular stability.

Map the causal pathway from variant to phenotype explicitly: variant to protein dysfunction to cellular phenotype to tissue remodeling to clinical manifestation. Each link must be supported by evidence and be mechanistically plausible. Anchor this mapping in established mechanistic literature [9,11,26]. Identify where the chain becomes inferential and where compensatory mechanisms may obscure primary drivers.

Evaluate whether the pathway node exhibits consistent perturbation across independent human datasets. Apply the Molecular Concordance Scoring Matrix (Table 3) across five domains: directional agreement (C1), effect size consistency (C2), baseline variability ratio (C3), genetic anchoring (C4), and model recapitulation (C5). Each domain is scored 0 to 2, yielding a maximum of 10 points. The three decision categories introduced in the “Molecular concordance as a translational gate” section apply directly: scores of 8 to 10 trigger an Advance decision; scores of 5 to 7 trigger a Conditional Advance requiring a prospectively defined validation plan before phase II initiation; scores below 5 trigger a Do Not Advance decision requiring orthogonal validation before reentry. These thresholds are conceptual rather than empirically calibrated against historical development outcomes; future validation against a curated dataset of cardiomyopathy programs would permit data-driven refinement. The act of scoring forces explicit documentation of stability evidence before advancement decisions are made; its primary value is discipline, not precision.

Incorporate baseline expression variability from healthy myocardium [19] as the benchmark for the C3 domain. RWE-derived phenotypic stratification can serve as an orthogonal validation layer at this step, where clinical data are available [6,27].

Select pharmacodynamic and stratification biomarkers that reflect the mechanistic node. Demonstrate reproducibility across sites and analytic pipelines [8,13]. Validate that the biomarker responds to modulation of the target before investing in it as a pharmacodynamic readout. Novel biomarkers must demonstrate not only mechanistic linkage but also practical stability under real-world collection and processing conditions.

Confirm that chosen preclinical systems replicate both the targeted mechanistic node and at least one downstream phenotype relevant to human disease [17]. Recognize and document model limitations, particularly regarding the maturation state of iPSC-CMs and the absence of multicellular architecture. Positive results in systems that do not capture the human mechanistic chain should be treated as exploratory rather than confirmatory.

Assess delivery, durability, reversibility, immunogenicity, and off-target risk according to therapeutic platform [28]. Mechanistic stability directly informs modality selection. A stable, mutation-proximal loss-of-function mechanism may justify durable genomic correction. A context-dependent signaling cascade may be better addressed with reversible small-molecule modulation. Misalignment between mechanism stability and modality irreversibility amplifies development risk substantially.

**Figure 2:**
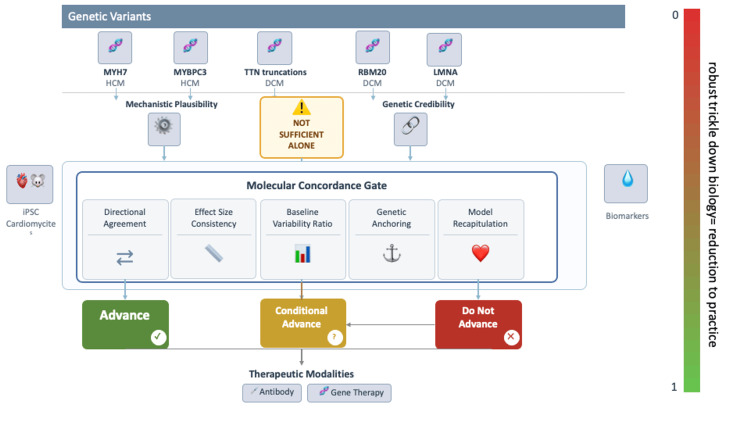
Molecular concordance gate framework for target validation in genetic cardiomyopathy Representative cardiomyopathy genes (MYH7, MYBPC3, TTN truncations, RBM20, and LMNA) feed into the framework through two conventional inputs, such as mechanistic plausibility and genetic credibility, which are necessary but not sufficient alone for advancement. The Molecular Concordance Gate integrates five scored domains: directional agreement (C1), effect size consistency (C2), baseline variability ratio (C3), genetic anchoring (C4), and model recapitulation (C5). Gate output determines one of three development decisions: Advance (high concordance, score 8-10), Conditional Advance (moderate concordance, score 5-7), or Do Not Advance (low concordance, score < 5). Approved targets proceed to therapeutic modality selection. DCM, dilated cardiomyopathy; HCM, hypertrophic cardiomyopathy; iPSC-CMs, induced pluripotent stem cell-derived cardiomyocytes This figure was created by the authors using Microsoft PowerPoint.

Establish predefined quantitative thresholds for molecular concordance score, target engagement, biomarker reproducibility, and safety margins. This final step transforms mechanistic insight into actionable development criteria and replaces narrative persuasion with structured evaluation. Go/no-go criteria must be defined prospectively, before data are examined, to prevent post hoc rationalization of advancement decisions. The scoring criteria across all five domains are presented in Table 4. The Molecular Concordance Gate framework, showing how these domains integrate to produce advancement decisions, is illustrated in Figure 2. The full seven-step operational summary is presented in Table 5. Table 6 situates this framework relative to current translational practice, identifying what existing approaches lack and what this framework adds.

**Table 4:** Molecular concordance scoring matrix: five-domain assessment for translational target prioritization Each domain (C1-C5) scored 0, 1, or 2. Maximum score = 10. ClinVar P/LP, pathogenic or likely pathogenic; CV, coefficient of variation; GTEx, Genotype-Tissue Expression Consortium; iPSC, induced pluripotent stem cell

Domain	High (score = 2)	Moderate (score = 1)	Low/unstable (score = 0)
C1: Directional agreement	Consistent direction in three or more independent human cohorts	Consistent in two cohorts; divergent in one or more	Inconsistent direction across two or more cohorts
C2: Effect size consistency	CV of effect sizes 30% or less across cohorts	CV 30-60%; notable cohort-specific variation	CV above 60%; or effect size near zero in one or more cohorts
C3: Baseline variability ratio	Disease signal exceeds 2x normal SD (GTEx benchmark)	Disease signal 1-2x normal SD	Disease signal falls within normal SD range
C4: Genetic anchoring	Direct variant-to-pathway link; ClinVar P/LP with functional evidence	Plausible mechanistic link; limited functional confirmation	Pathway association only; no direct genetic linkage
C5: Model recapitulation	Pathway node reproduced in two or more validated human-relevant systems	Reproduced in one system; partial phenotypic alignment	Animal model or immature iPSC only; no phenotypic alignment
Total score	8-10: High stability - Advanced	5-7: Moderate - Conditional advance with validation plan	0-4: Unstable - Do not advance without orthogonal validation

**Table 5:** Seven-step translational framework: operational summary Each step corresponds to a specific translational failure mode identified in the genetic cardiomyopathy development literature. PD, pharmacodynamic

Step	Gate	Key question	Failure mode addressed
1	Genetic credibility	Is the variant robustly linked to disease with functional evidence?	Pursuing variants of uncertain significance
2	Mechanistic chain	Is the causal path from variant to clinical phenotype fully specified at each step?	Targeting epiphenomenal or compensatory nodes
3	Cross-cohort concordance	Does the pathway signal replicate consistently across independent human datasets? (apply Table 3 scoring)	Cohort-specific false positives; biological instability undetected
4	Biomarker alignment	Are PD and stratification biomarkers mechanism-linked and reproducible across sites?	Unreliable dose-response interpretation; trial enrichment failure
5	Model fidelity	Does the preclinical system recapitulate the human mechanistic node and at least one downstream phenotype?	Model-specific artifacts that fail to translate to humans
6	Modality feasibility	Is the therapeutic platform appropriate, given the stability and reversibility profile of the mechanism?	Irreversible genomic intervention on context-dependent target
7	Go/no-go criteria	Are quantitative advancement thresholds defined prospectively before data are examined?	Post hoc rationalization of advancement despite insufficient concordance evidence

**Table 6:** What the field currently uses, why it is insufficient, and what the framework adds Reference numbers are provided for the current practices cited. The framework additions are described in detail in the “Reproducibility, molecular stability, and development risk” and “A structured translational framework for genetic cardiomyopathy development” sections. GTEx, Genotype-Tissue Expression Consortium; iPSC-CMs, induced pluripotent stem cell-derived cardiomyocytes

Current practice	Why it is insufficient	What this framework adds
ClinVar pathogenicity annotation [6]	Genetic association does not imply molecular stability. A variant robustly linked to disease may not produce consistent downstream transcriptomic or proteomic consequences across patient cohorts.	Explicit cross-cohort concordance assessment as a scored gate (C1-C3 domains) between genetic credibility and advancement decision.
Single-cohort transcriptomic profiling [13]	Signals identified in one cohort frequently do not replicate. Cohort composition, disease stage, medication exposure, and platform differences generate dataset-specific artifacts that are indistinguishable from true biology without cross-cohort comparison.	Prespecified multi-cohort concordance scoring with GTEx-benchmarked baseline variability as a reference, preventing conflation of technical or context-specific signal with universal disease biology.
iPSC-CM or animal model support [10,17]	Preclinical models may fail to capture human isoform context (alpha- versus beta-myosin heavy chain), developmental maturity, or multicellular remodeling dynamics, leading to model-specific confirmation that does not translate.	Model fidelity as a formally scored domain (C5), requiring reproduction of the targeted mechanistic node in at least one human-relevant system and documentation of model limitations before confirmatory weight is assigned.
Biomarker correlation studies [8,13]	Biomarkers that correlate with disease severity may be reactive rather than causally linked to the therapeutic target, making them unreliable pharmacodynamic readouts in the context of a specific mechanism-directed intervention.	Biomarker-mechanism alignment as Step 4, requiring explicit demonstration that the biomarker responds to modulation of the target and maintains reproducibility across sites and analytic platforms.
Mechanistic plausibility alone [11,26]	Plausibility establishes a hypothesis. It does not confirm that the mechanism operates consistently at the level of the proposed intervention across the biological diversity of patient populations. Context-dependent mechanisms can be plausible and simultaneously unstable.	Stability-driven advancement decision as the organizing principle: mechanistic plausibility is necessary but not sufficient. A concordance score must accompany the plausibility argument before the framework permits advancement.

Clinical trial enrichment and regulatory implications

Clinical trials in genetic cardiomyopathies face a fundamental paradox. The diseases are genetically defined, yet phenotypically heterogeneous. Carriers of identical sarcomeric mutations may exhibit divergent trajectories influenced by modifier genes, environmental exposures, and metabolic status [2]. This heterogeneity can dilute the therapeutic signal. If a mechanistic node is active only in a subset of patients, enrolling an unstratified population risks systematically underestimating the true treatment effect, a problem particularly acute for therapies targeting downstream pathways such as fibrosis or inflammatory signaling that may be stage- or context-dependent [12,13].

RWE and electronic health record (EHR)-derived datasets offer a complementary and underutilized dimension for concordance validation in genetic cardiomyopathy [27]. While clinical trials provide mechanistic purity, RWE captures the biological heterogeneity of real patient populations, such as variable comorbidity burden, concomitant pharmacotherapy, metabolic status, and longitudinal disease trajectory. Machine learning-powered risk calculators built on EHR data can quantify the baseline frequency of adverse events, stratify patient subgroups by pathway-relevant biomarkers, and identify modifier interactions that single-cohort transcriptomic analyses miss. In the context of molecular concordance assessment, RWE-derived phenotypic stratification provides an orthogonal validation layer: if a therapeutic target exhibits concordant transcriptomic behavior across independent Gene Expression Omnibus datasets but the corresponding phenotypic endpoint does not show a consistent signal in RWE cohorts, this discordance should function as a developmental caution flag rather than being attributed to trial design variability. Integrating RWE-based adverse event risk profiling into the concordance scoring framework at Step 3 strengthens the translational evidence package and aligns with regulatory expectations for multi-source validation in genetic disease programs [6].

The success of sarcomere modulation in obstructive HCM offers an instructive contrast. Hypercontractility is phenotype-proximal and consistently expressed in obstructive disease [5,10]. Enrichment based on obstruction and symptomatic status contributed to reliable signal detection in the EXPLORER-HCM trial. Future trials targeting less stable downstream nodes may require molecular concordance-based stratification, baseline biomarker thresholds indicating pathway activation, polygenic risk-informed subgroup analyses [2], and stage-specific enrollment criteria. Trial design must integrate stability assessment upstream of randomization, not after enrollment failures reveal it.

**Figure 3:**
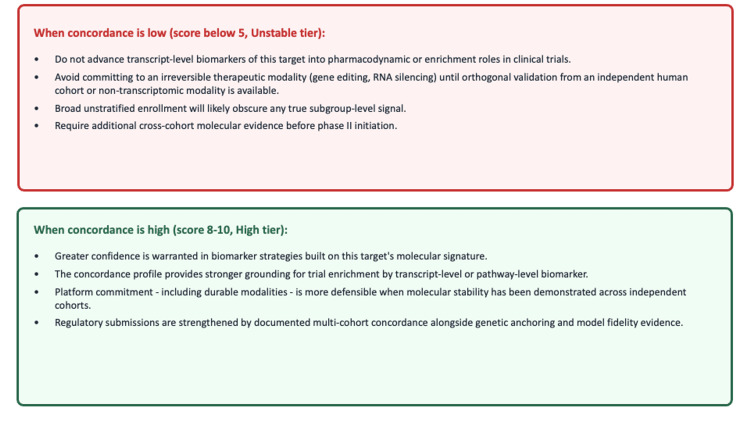
Clinical translation implications of concordance tier assignment The figure presents two concordance-tier decision boxes. The upper box (red border) specifies four action constraints when concordance is low (score below 5, Unstable tier). The lower box (green border) specifies four actions enabled when concordance is high (score 8-10, High tier). This figure was created by the authors using Microsoft PowerPoint.

Regulatory agencies increasingly emphasize biomarker qualification and mechanism-based development pathways. For a biomarker to serve as an enrichment or pharmacodynamic tool, reproducibility across sites and analytic platforms is critical. Exosomal biomarkers exemplify the regulatory challenge: isolation protocols vary, normalization standards are evolving, and AI-driven classification may introduce hidden biases [8]. GTEx-derived baseline variability data provide an additional benchmarking tool [19], and demonstrating that a disease-associated signal exceeds normal interindividual variance strengthens regulatory credibility. Different therapeutic modalities carry distinct regulatory burdens; mechanistic stability directly informs modality choice, and regulatory confidence is strengthened when genetic anchoring, cross-cohort concordance, biomarker reproducibility, and model human fidelity can all be affirmatively demonstrated [17,28]. The clinical translation implications of concordance tier assignment are summarized in Figure 3.

Payer implications

From a health economics and outcomes research perspective, the framework has important implications for precision medicine pricing and reimbursement. Molecular concordance effectively defines the size and stability of the treatable population, which is a key driver of value-based pricing models. Targets with high molecular stability across genetically defined populations are more likely to produce consistent treatment effects, enabling broader indications, more predictable outcomes, and pricing models based on population-level value. In contrast, targets with low concordance may only work in molecularly defined subgroups, implying smaller treatable populations, the need for companion diagnostics, and pricing models aligned with high-value, small-population precision therapies similar to gene therapies or oncology biomarker-driven drugs. Therefore, molecular concordance scoring could function not only as a translational development gate but also as an early indicator of commercial strategy, informing whether a therapy should be priced as a broad cardiology therapy, a stratified precision medicine therapy, or an ultra-targeted rare disease intervention. In this sense, molecular stability is not only a scientific variable but also a pricing and reimbursement variable, because it determines population size, response predictability, payer risk, and ultimately value-based pricing frameworks.

Discussion

Genetic cardiomyopathies occupy a unique position in cardiovascular medicine. They are among the best-characterized inherited cardiac disorders at the genetic level, yet they continue to challenge translational execution. Decades of research have clarified the molecular architecture of sarcomeric dysfunction, cytoskeletal instability, calcium signaling, and maladaptive remodeling [3,10]. At the same time, the field has experienced selective breakthroughs, with sarcomere modulation in obstructive HCM [5] being the clearest example, contrasted by stalled or inconsistent programs targeting upstream signaling pathways. We argue that the limiting factor is no longer gene discovery or pathway identification but the absence of structured evaluation of molecular stability, cross-cohort reproducibility, and mechanistic alignment prior to clinical advancement.

The Novelty of This Framework

The cardiomyopathy literature has addressed molecular mechanisms in depth [10,26], catalogued novel biomarkers and multi-omics discoveries [13], and surveyed therapeutic modalities from small molecules to gene editing [28]. What has been absent is a framework that formally positions cross-cohort molecular concordance as a scored, development-ready gate, a criterion that must be satisfied before mechanistic plausibility is permitted to advance a target. The contribution here is not new biology. It is discipline: the requirement that stability be assessed explicitly, documented prospectively, and treated as a property of the mechanism rather than a statistical quality check applied after the fact.

The matrix is one implementation of this principle. Development teams in other disease contexts may adapt domain weights, thresholds, or evidence criteria to their setting. The principle that concordance must be affirmatively demonstrated rather than assumed remains binding regardless of implementation. Table 2 demonstrates how the framework organizes existing published evidence for representative targets without requiring new data, and Table 5 situates this contribution relative to current norms.

Stability as a Translational Variable

Mechanistic plausibility has served as the primary advancement gate in therapeutic prioritization, but plausibility does not equate to stability. Stability determines whether a mechanism will withstand the biological diversity of patient populations encountered in clinical trials. HCM and DCM, despite being described as monogenic disorders, are shaped by common variant backgrounds, modifiable risk factors, and environmental exposures that substantially influence expressivity [2]. TTN truncations demonstrate incomplete penetrance and variable severity [4]. Failure to stratify by disease stage, genetic modifier burden, or mechanism class when interpreting molecular evidence risks advancing targets that are cohort-specific rather than disease-defining. The concordance gate exists to force this confrontation upstream, before development commitments are made.

Multi-Omics and AI: Calibrating Enthusiasm With Rigor

Multi-omics integration has expanded the candidate target landscape dramatically. Proteomic, metabolomic, and circulating biomarker studies identify novel mechanistic nodes and refine disease phenotyping [13]. AI workflows promise integrative prioritization across high-dimensional datasets [24]. Yet these approaches introduce fragility. Feature selection can be cohort-specific. Exosomal cargo profiles are sensitive to isolation protocols and batch effects [8]. Without transparent validation and external replication, sophisticated analytic pipelines may produce impressive internal metrics while lacking generalizability. The concordance scoring framework provides a structured means of evaluating AI-derived targets against the same stability criteria applied to classical pathway targets, ensuring that algorithmic discovery is held to the same reproducibility standards as conventional mechanistic research.

Model Fidelity and Platform Alignment

Preclinical systems remain indispensable but imperfect. Species differences in contractile protein expression and electrophysiology complicate translation [10]. iPSC-derived cardiomyocytes offer a human genetic context but require maturation to approximate adult physiology [17]. A mechanistically complete validation package must demonstrate alignment across human genetic evidence, human molecular datasets, fit-for-purpose models, and biomarkers intended for clinical use. Platform selection interacts critically with mechanism stability: small molecules offer reversibility and titration, while RNA-based therapies and gene editing introduce durability but increase regulatory and safety complexity [28]. Modality choice should be driven by mechanistic context rather than technological enthusiasm.

Limitations

Several limitations merit consideration. First, heterogeneity in published datasets restricts direct quantitative comparison across studies. Second, publication bias may overrepresent positive mechanistic findings, potentially inflating perceived target robustness. Third, emerging modalities such as genome editing and regenerative reprogramming are evolving rapidly, and long-term safety profiles remain incompletely characterized. Fourth, the concordance scoring thresholds proposed in Table 3 are conceptual rather than empirically calibrated; validation against development outcomes across cardiomyopathy programs would strengthen their utility and permit threshold refinement. Fifth, this work does not represent a meta-analysis and does not provide pooled quantitative estimates; heterogeneity in study design, tissue sources, analytic pipelines, and patient populations limits direct comparison across datasets. Nonetheless, structured validation remains preferable to implicit assumption, and these limitations do not undermine the core argument for concordance as a translational gate.

What Evidence Would Validate This Framework?

The framework presented here is conceptual. Its scoring domains, threshold values, and advancement decision rules are grounded in mechanistic reasoning and translational precedent, but they have not been empirically calibrated against historical development outcomes. Prospective validation is, therefore, a necessary next step and is explicitly acknowledged as such.

Validation would require, at minimum, the following evidence base. First, independent multi-cohort human transcriptomic analyses applying the concordance scoring matrix to ClinVar-annotated cardiomyopathy genes across publicly available datasets would test whether the scoring instrument produces stable, interpretable tier classifications and whether those classifications align with mechanistically motivated expectations (e.g., lower concordance for sarcomeric contractile genes than for RNA-processing mechanism genes). Second, GTEx-benchmarked baseline variability quantification for each scored gene would confirm that the C3 domain discriminates disease signal from normal interindividual variation as theorized. Third, prospective threshold calibration against a curated dataset of cardiomyopathy development programs with known outcomes, such as advancing, stalling, or failing at each stage, would permit data-driven refinement of the 5-7 and 8-10 score cutoffs proposed in Table 3. The general principle that scoring instruments require empirical calibration against historical outcomes before their sensitivity and specificity can be characterized has been established in the context of preclinical replicability assessment [29].

Fourth, disease-generalization testing in DCM, arrhythmogenic cardiomyopathy (ARVC), and potentially non-cardiac Mendelian disorders with well-characterized transcriptomic datasets would examine whether the framework’s mechanistic stratification principle holds across pathogenic mechanism classes beyond the HCM context in which it was developed. Fifth, concordance-outcome linkage studies, which examine whether genes classified as High concordance tier by the matrix produce more reliable pharmacodynamic signals in subsequent clinical programs than genes classified as Unstable, would constitute the most direct empirical validation of the framework’s clinical utility.

Until this evidence is available, the framework should be applied as a structured reasoning aid that forces explicit documentation of stability evidence, rather than as a validated predictive instrument with calibrated sensitivity and specificity. The value of its current form lies not in the precision of its scores but in the discipline it imposes on advancement decisions.

Broader Implications Beyond Cardiomyopathy

Genetic cardiomyopathy is the exemplar chosen here because it offers a rare combination: strong genetic anchoring, deep mechanistic characterization, multiple mechanism classes within a single disease family, and a documented history of translational failures that cannot be explained by insufficient preclinical evidence. These properties make it an ideal context in which to develop and illustrate the concordance principle. The principle itself, however, is not cardiomyopathy-specific.

The same gap between genetic credibility and molecular stability appears in any genetically anchored disease where the pathogenic mechanism operates at a level removed from transcript abundance. Long QT syndrome variants in SCN5A and KCNQ1 alter ion channel function at the protein level [30]; transcript-level biomarker strategies for these genes carry the same conceptual risk as those for MYH7. ARVC, driven by desmosomal gene variants including plakophilin-2 and desmoplakin, involves intercalated disc disruption and fibrofatty replacement whose transcript-level signatures are heterogeneous across published cohorts [31]. Trigger-associated cardiomyopathies extend this principle further: transcriptome analysis of peripartum cardiomyopathy has identified proteostasis and cell survival pathway disruption as disease-specific signatures [32], demonstrating that environmental triggers such as pregnancy produce molecularly distinct cohort profiles whose transcript-level concordance cannot be assumed across unselected DCM populations. Mendelian kidney disorders, inherited channelopathies, and connective tissue diseases all contain gene classes where protein-level mechanisms dominate while RNA-level evidence remains heterogeneous across cohorts. The broader implication is that genetic credibility and mechanistic plausibility together define a necessary but not sufficient evidence package for translational advancement in any genetically complex disease. Stability evidence, graded by the concordance framework or an adapted equivalent, represents the missing third pillar. Making this pillar explicit and scored, rather than implicit and assumed, is a principle the field can generalize well beyond cardiomyopathy.

Methodological Fragility and Publication Bias

While the Molecular Concordance Scoring Matrix offers a standardized framework, the underlying data is subject to inherent methodological fragility. Variability in tissue procurement (e.g., end-stage explant vs. biopsy) and the "snapshot" nature of transcriptomic data can obscure dynamic stability processes. Furthermore, a discernible publication bias exists toward positive protein-transcript correlations, which may underrepresent the frequency of discordant translational gates in less-studied cardiomyopathy variants. Acknowledging these limitations is essential for accurately calibrating the risk of bias in early-stage drug discovery. 

Future Directions: AI and Multi-Omics

The integration of AI-driven discovery and multi-omics has recently refined our understanding of these gates. Recent studies [33,34] have utilized large-scale transcriptomic datasets to predict protein folding and stability with unprecedented accuracy, further validating the need for the concordance framework proposed in this review.

## Conclusions

The dominant translational error in genetic cardiomyopathy is the advancement of targets based on pathogenicity annotation and mechanistic plausibility alone, without structured evaluation of whether molecular consequences are stable and reproducible across independent patient cohorts. Molecular stability is a property of mechanism, not of genetic evidence, and cross-cohort concordance must serve as an explicit development gate alongside plausibility. The seven-step framework and Molecular Concordance Scoring Matrix presented here operationalize this principle in one implementable form. The field has the genetic clarity and mechanistic depth to demand concordance as a standard preclinical gate. What is needed now is the discipline to apply it.
